# Michael J Clague: mitophagy, pexophagy, & metabolism

**DOI:** 10.26508/lsa.202302102

**Published:** 2023-04-26

**Authors:** Michael J Clague

**Affiliations:** Department of Molecular Physiology and Cell Signalling, Institute of Systems, Molecular and Integrative Biology, University of Liverpool , Liverpool, UK

## Abstract

Michael J Clague is a Professor of Molecular and Cellular Physiology at The University of Liverpool, Institute of Systems, Molecular and Integrative Biology. We asked him about his recent paper published in Life Science Alliance (LSA) and his experience in science thus far.

## Abstract (precis)

Interview with Michael J Clague

**Figure 1:**
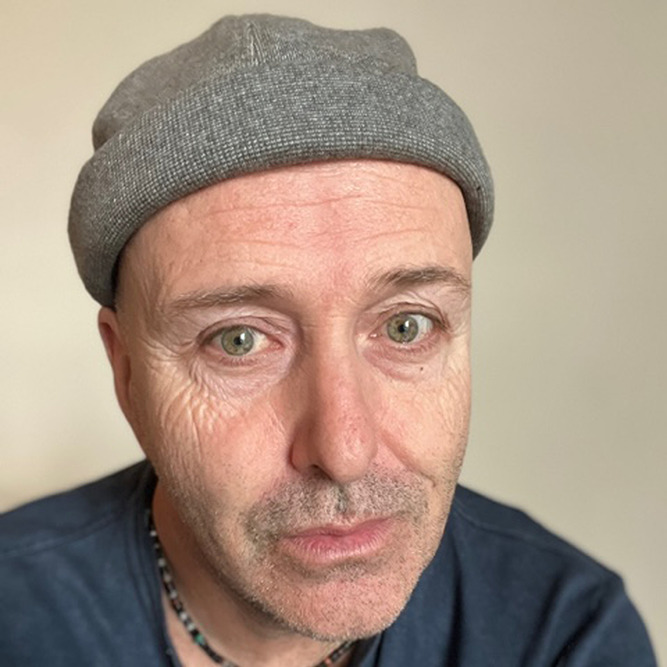
Michael J Clague

## How would you explain the main findings of your article, and how did It come about?

We have been studying the selective autophagy of mitochondria (mitophagy) for some time. We have a particular interest in a mitochondria-localised deubiquitylase (DUB), USP30, which is an actionable target for Parkinson’s disease. Loss or inhibition of USP30 can promote PINK1-dependent mitophagy. Some time ago, we discovered that a small fraction of USP30 is on peroxisomes and its loss could promote pexophagy, albeit in a PINK1-independent manner. This heightened our awareness of the overlap in membrane components between mitochondria and peroxisomes and of their alliance in controlling aspects of metabolism.

The PINK1/Parkin pathway is of such intense interest that many people are surprised to learn that it is not the major pathway leading to mitophagy in living organisms. This was first shown by Ian Ganley using a mouse model and us, together with Alex Whitworth, using fly models bearing mitophagy reporters (1, 2). More recently, we conducted a CRISPR/Cas9 screen of ubiquitin E3 ligases for regulators of mitophagy and found that the Cullin RING E3 ligase (CRL) components VHL and FBXL4 were very strong suppressors. Both of these converge on determining levels of the mitophagy adaptor NIX: VHL via its transcription and FBXL4 via destabilisation. We could recapitulate these findings by using a general inhibitor of Cullin RING ligase activity, MLN2924, to promote mitophagy (3). Thus, NIX was very much on our mind and it became obvious to test for a role in pexophagy.

Most of the treatments that acutely induce pexophagy are rather nonspecific or poorly defined. We could show that MLN2924 efficiently induced pexophagy, close to the level seen with an iron chelating drug (DFP) which similarly elevates NIX via HIF1α up-regulation. Depletion of NIX confirmed this mechanism by returning pexophagy levels to baseline. We also found that a highly specific USP30 inhibitor, Compound 39, could also induce pexophagy, not quite as well, but within the same ballpark as DFP. What is interesting is that this pathway is NIX independent, but rather depends on the ubiquitin-binding adaptor molecule NBR1. Our article offers new ways to acutely induce pexophagy and highlights their coupling to mitophagy (4).

## What was the decision process in choosing where to publish?

We have two previous publications describing USP30 inhibitors published in LSA and had been very satisfied with the editorial process (5, 6). This article felt like part of that series and we could be confident that the editorial team would recognise the fit to the journal’s remit. Another consideration was that whilst our manuscript was in preparation, the Ganley laboratory also published a role for NIX in pexophagy after the application of DFP (7). Our work is complementary to that study and we wanted to find a journal which would be comfortable with that partial overlap. Given a choice, we favour publication in nonprofit journals. It is also important that the journal has a reputation for fairness alongside rigour.

## What advice do you have for other researchers on maximizing the dissemination of their work?

Take care with the title of any publication. I have sometimes not thought hard enough about this and maybe one or two articles have fallen through the cracks as a consequence. Stay patient and make your article the best it can be. I am a strong advocate for making preprints available before publication, for example on bioRxiv.

## What questions is your laboratory currently trying to answer?

My laboratory is codirected with Sylvie Urbé and our combined input allows us to cover a wider area than might be usual. One theme that has emerged in the last few years is the cell biology of Parkinson’s disease (PD). We are seeking to make the mechanistic connections between various PD genes. I have set forth a kind of slogan that “if cancer is a pathology of cell signaling, Parkinson’s disease is one of membrane trafficking.” PD genes converge around the preservation of mitochondrial and endolysosomal health and this falls right within our central interests. It feels like we should be able to make good contributions in that field in the coming years. I also find Michael J Fox an inspiring individual, and his foundation is the most impressive charitable funder I have worked with. I think some other aspects of mitochondrial biology will also keep us occupied for a while. We will work on further understanding FBXL4 control of NIX stability and we have developed an interest in the mitochondrial import (TOMM) complex. Our oncology interests are currently confined to understanding the endosomal trafficking of receptors linked to immune checkpoint regulation. Finally, we have a couple of new methods under development that we hope to publish shortly.

## What motivated you to pursue a career in science, and what have been the most interesting moments on the path that led you to where you are now?

I felt that I had some talent at it and that made me curious to put that notion to the test. My first turning point was progressing from undergraduate chemistry to postgraduate membrane biophysics. Later, I wanted to see the world and the itinerant post-doc life was very appealing. I continued along the vein of membrane biophysics at NIH. I was flattered and exhilarated by the positivity of the US mindset and became immediately engaged in many fruitful collaborative studies. I have always been a keen reader of the literature, but generally stayed in my lane at that point. There was a small journal club that met a few floors above me, normally attended by about 20 or so people. Critically, it was devoted to discussing new advances in cell biology. I had been studying enveloped virus fusion mechanisms, but now had somehow drifted into a room discussing work from Jim Rothman and colleagues proposing a “universal mechanism of intracellular membrane fusion.” This immediately captured my imagination and I decided that this was going to be the field I pursued next. “Where can I learn cell biology?” I asked, without any real clue. I got the advice to go to EMBL in Heidelberg, Germany, and managed to get a long-term EMBO fellowship to carry out work in their famous Cell Biology Programme. It was one of the crucibles of what we would call “membrane trafficking” research. It was there that I got my education in cell biology and I was a pretty quick learner. I came out of EMBL much harder boiled and ready to start my own group.

On starting my laboratory in Liverpool, I carved out a niche investigating the interplay between endocytic signaling and receptor trafficking, particularly the role of phosphatidylinositides. Under the encouragement of the late George Vande Woude, we began to study the Met receptor and this led us to ubiquitin biology. Attending my first conference on ubiquitin in Colorado, I came away with the feeling that there was space for some more cell biology. It was also apparent to me at that time that there was much more focus on the conjugation pathway than deconjugation by DUBs. I smelt an opportunity, and working together with Sylvie Urbé, we were able to characterise two endosome-associated DUBs and show how these could influence the fate of activated receptors. After this early success, we seized our opportunity by building a platform to study DUB function more widely. I compare it with going into a casino and putting everything we had on red, except we bet on DUBs. We had now found a theme that was scalable and could be pursued over the long term.

I think we did also have a vision, even then, that DUBs might be good drug targets. We advocated for this in a couple of reviews and all of a sudden, we began to get approaches from industry to visit and advise. Skeptical at first, I have come to enjoy these interactions and it now gives an extra flavour to our laboratory. We have worked with a start-up, multiple SMEs, and Big Pharma. As part of this, we have been involved in studies that introduced the first highly selective DUB inhibitors that truly helped move the field forward. It is gratifying to see that such inhibitors are now entering clinical trials this year. More recently, the idea to leverage DUB activity by induced proximity (kind of the opposite to PROTACs) has gained traction and we have been involved with a new start-up that seeks to lead in this area.

## Tell us something interesting about yourself that would not be on your CV

I asked the person who knows me best how I might answer this and they told me “the most interesting thing about you is what you are not interested in.” I am able to free up enough headspace to explore my interests in cinema, music, and politics, which tend to be a bit off the beaten track. I think it is important to have a hinterland. The eminent Parkinson’s disease researcher Andrew Lees writes about this in his memoir “Mentored by a Madman.” He attributes his introduction of apomorphine into the PD clinic, at least in part to his interest in the “beat writer” William S Burroughs, who was treated with this drug whilst trying to come off heroin. I do not have such a direct example, but diverse interests and experiences provide a well to draw on.
